# An Unusual Pattern of Scapular Avulsion Fracture in the Spine: A Case Study

**DOI:** 10.7759/cureus.62132

**Published:** 2024-06-11

**Authors:** Ashwin Deshmukh, Ishan Shevate, Rahul Salunkhe, Archit Gupta, Shubhankar Chopra

**Affiliations:** 1 Orthopaedics, Dr. D. Y. Patil Medical College, Hospital and Research Centre, Dr. D. Y. Patil Vidyapeeth, Pune (Deemed to Be University), Pune, IND

**Keywords:** sports related injuries, conservative approach, atypical presentation shoulder pain, scapula fracture, acromion process fracture

## Abstract

Acromion fractures, particularly isolated cases, are uncommon but significant in shoulder injuries. There is no universally accepted treatment protocol, but the classification of the fracture helps to guide clinical decisions. We present a case report aiming to contribute to the understanding of treatment options for acromion fractures. A 22-year-old male sustained a left shoulder injury during a wrestling match, resulting in a type 2 acromion fracture. Conservative treatment was initiated with regular follow-ups. Serial imaging showed no further displacement. Gradual rehabilitation exercises were introduced based on healing progress. The rarity of isolated acromion fractures complicates their management. Conservative management, coupled with rehabilitation exercises, yielded positive outcomes in our case, suggesting its efficacy as a primary treatment option for isolated displaced acromion fractures. Further research is needed to establish standardized protocols for managing such fractures, but until then, conservative care remains a viable approach, potentially preferred over surgical intervention.

## Introduction

The anteriorly extending lateral projection of the scapula spine is called the acromion process. Scapular fractures are infrequent injuries, accounting for up to 3% of all shoulder fractures, but isolated acromion fractures constitute only 9% of all scapular fractures [1]. Acromial fractures typically result from superior dislocation of the humeral head or direct trauma to the shoulder. There is no general universally accepted treatment plan. Still, for categorization, there are three categories of acromial fracture defined by Kuhn et al. [2] that can be used to assess whether surgical or non-surgical treatment might be appropriate: Type I involves un-displaced fractures, including IA avulsion fractures or IB true fractures. Type II comprises displaced fractures that do not reduce the subacromial space. Type III entails displaced fractures resulting in a reduced subacromial space, which could result from an inferior acromion displacement or from a superiorly displaced glenoid neck fracture [1].

Historically, nonoperative intervention and early range of motion (ROM) have resulted in predictable healing, return to activities of daily living, and reduction in pain [2]. Because of the extensive muscular attachments and envelope, early and complete healing occurs. The large arc of scapular-thoracic and glenohumeral motion compensates for most deformities. Insufficient bone stock, complex three-dimensional anatomy, and difficult surgical exposures create problems with internal fixation [3].

The aim is to present a case report that contributes to the treatment plan for acromion fractures, and our case study aimed to demonstrate the functional result of conservative management for 12 weeks, followed by non-operative treatment of an isolated displaced acromion process fracture.

## Case presentation

**Figure 1:**
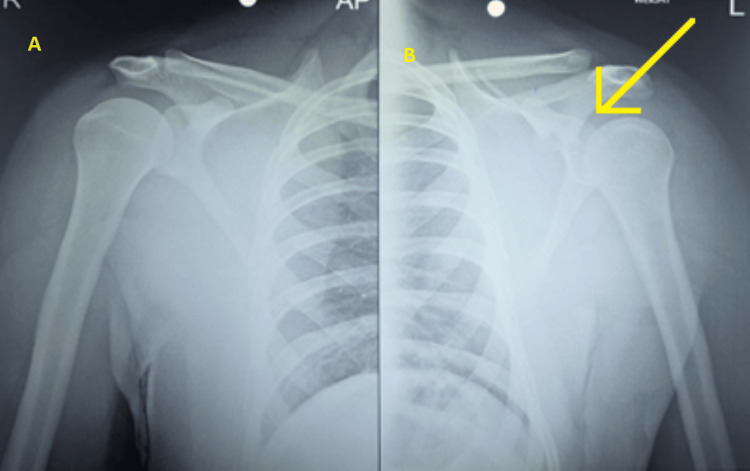
Plain radiograph at the time of presentation A) Right shoulder; B) Left shoulder The yellow arrow shows the fracture of the left acromion

**Figure 2:**
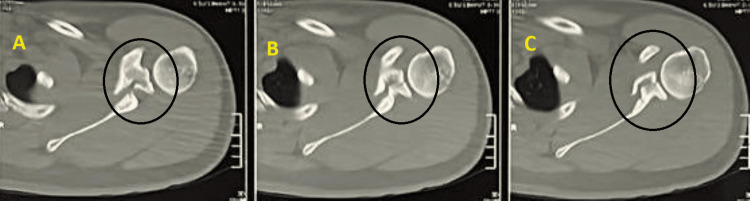
CT scan of the left shoulder joint A)-C) Serial axial cuts of the left shoulder joint The black circle shows the fracture site

A 22-year-old male arrived at the emergency room after he sustained the injury during a professional free-style wrestling match causing direct trauma in the left shoulder. He had crepitation and deformity in his left arm with no neurovascular deficit. A plain radiograph was obtained (Figure 1). The rotator cuff was intact. Based on the Kuhn classification, the acromion fracture found on the shoulder area CT scan was categorized as type 2 (Figure 2) [4].

At the time of presentation

**Figure 3:**
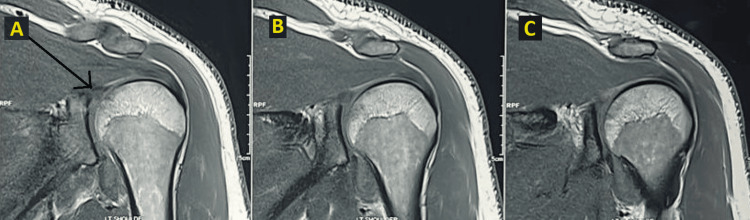
MRI of the left shoulder joint A)-C) Serial coronal MRI cuts of the left shoulder joint The black arrow shows the lesion

On the MRI, findings revealed a bony Bankart lesion accompanied by a labral tear, a glenoid fracture at the base of the coracoid, and a type 2 acromioclavicular joint injury (Figure 3).

It had been decided to treat the fracture along conservative lines, using a regular follow-up schedule. An arm bag and universal shoulder immobilizer were used for conservative treatment. Since these fractures are known to dislocate, serial radiological evaluation was obtained to check for indications of healing and, if any, displacement, after one week and then at three, six, 12, and 20 weeks.

Three Weeks After Conservation

**Figure 4:**
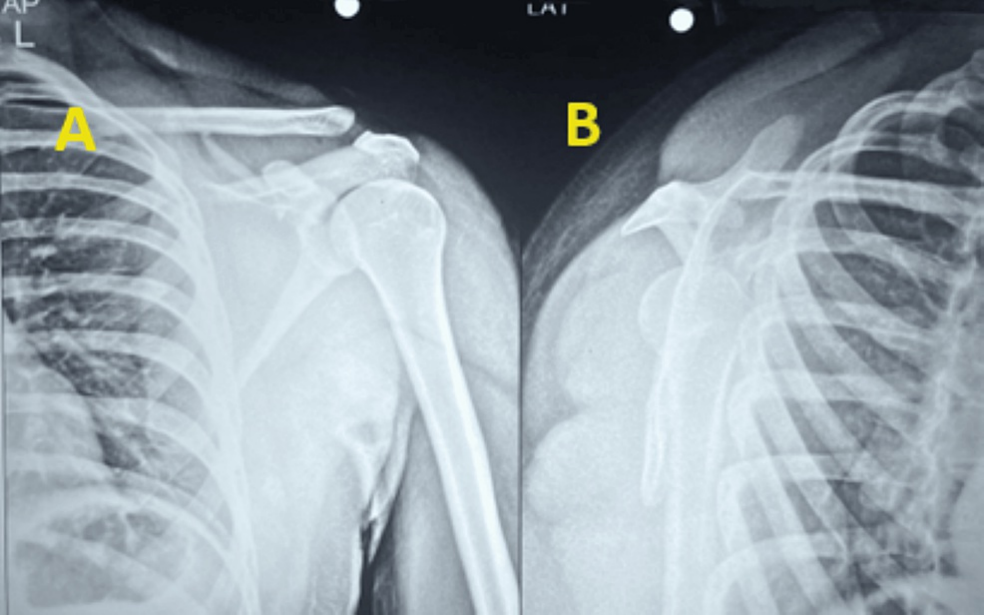
Plain radiographs showing anteroposterior view and lateral scapular view A) Anteroposterior view; B) Lateral scapular view

At the end of the three-week follow-up, there was evidence of a left-sided spine of scapula fracture. A fresh X-ray showed no further displacement of the fracture (Figure 4). The advice given was the gradual passive ROM of the left shoulder 0°-90° only, active elbow and wrist ROM exercises, and continuation of left shoulder ultrasound imaging (USI) for another three weeks.

Six Weeks After Conservation

**Figure 5:**
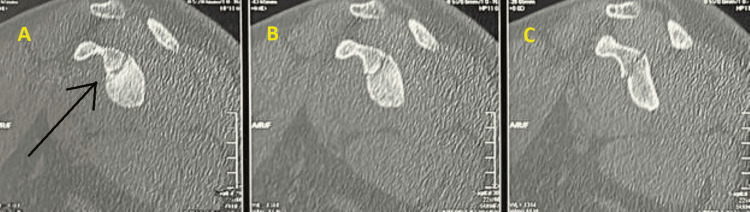
Repeat CT scan showing callus formation A)-C) Serial axial CT cuts of the left shoulder joint The black arrow shows callus formation

A repeat CT scan was done six weeks post-conservation which showed callus formation and no further displacement (Figure 5). The advice given was passive ROM exercises up to 90°-100° of the left shoulder.

Twelve Weeks After Conservation

Passive ROM assessment revealed that forward flexion from 0° to 150° was possible, but painful in the terminal arc. Abduction from 0° to 150° was possible which is normal. Therefore, the patient was advised to start assisted active shoulder ROM as tolerated with external rotation up to neutral.

Twenty Weeks After Conservation

**Figure 6:**
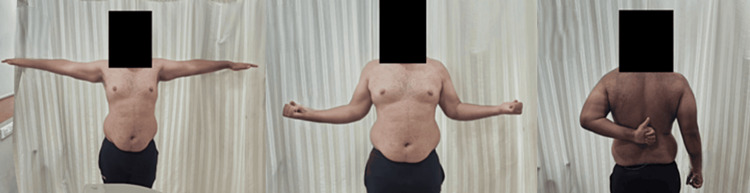
Post 20-week follow-up showing full range of movements achieved

**Figure 7:**
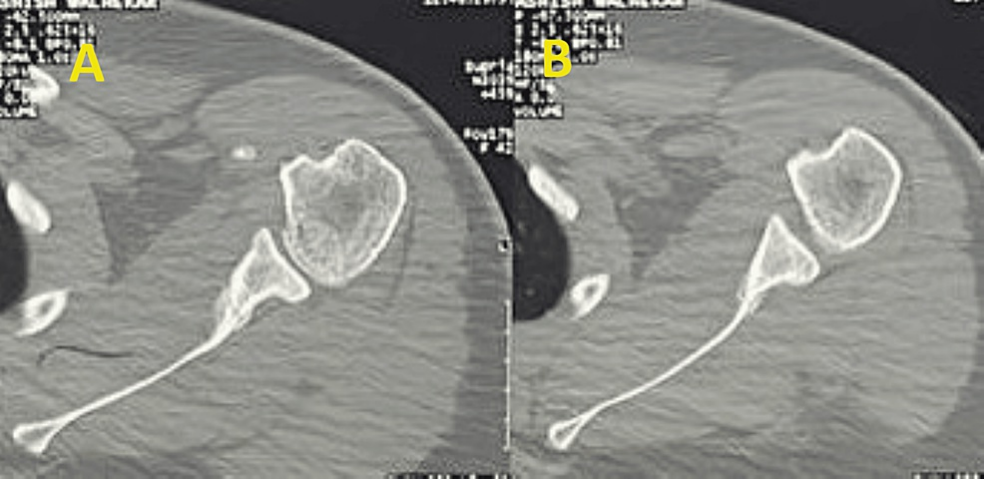
Post 20-week follow-up CT scan showing good callus formation and no further displacement A)-B) Serial axial CT cuts of the left shoulder joint

**Figure 8:**
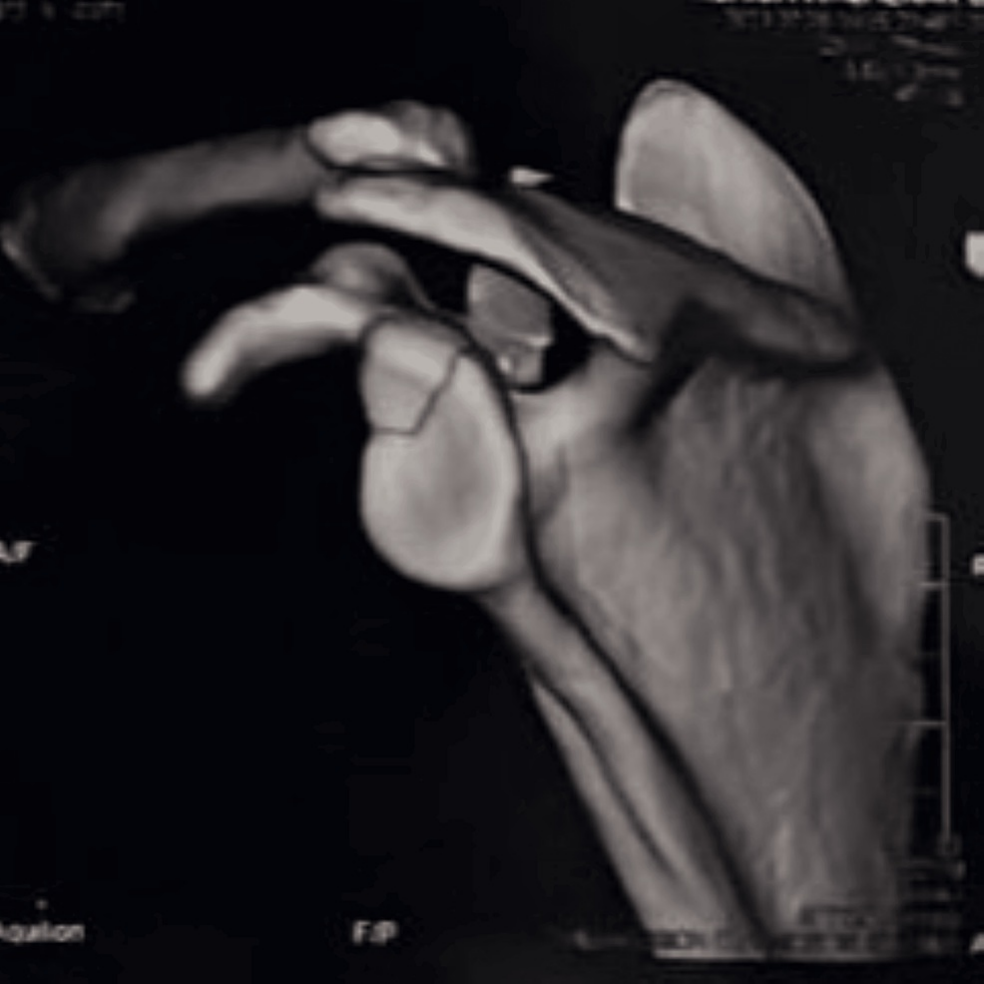
Post 20-week follow-up 3D CT scan

At this time, full ROM was achieved as shown in Figure 6. A repeat CT scan showed good callus formation and no displacement at the fracture site (Figures 7-8).

## Discussion

Anatomically located near the posterosuperior edge of the thorax, the scapula is linked to the pectoral girdle by many muscles and is a key component in the upper limb's biomechanics [5]. Acromion process fractures that occur alone after direct trauma are extremely rare. Currently, literature regarding the management of such fractures is lacking, partially because of hindrance by a lack of standardized techniques. Subacromial impingement, limited mobility, symptomatic non-union, pain, and rotator cuff injury can all arise from ill-treated acromion fractures [6].

Because the scapula is encased in a rich vascular network and muscular attachments, union rates should be high. Operative treatment strips soft tissue blood supply. The timing of postoperative radiographic imaging can influence the reported time to heal, and arbitrary points of three, six, eight, and 12 weeks may not be sufficient. Adiposity or ribs can obscure and interfere with the visualization of fracture union.

Many of these fractures have the propensity to heal uneventfully, numerous results have been reported for fractures that occur where the scapular spine and acromion base join. Our case had a fracture at this site. An AP, axillary, and radiological trauma series, along with MRI projections, are performed to assess any fractures in the shoulder region. Acromion process fractures can be classified, diagnosed, and treated more effectively with the use of a CT scan and should form an integral part of the diagnostic workup [7]. In our case, serial X-ray and MRI evaluations were performed every three weeks up to 20 weeks.

Most persons with type II AC injuries can resume their normal activities once their strength and ROM are fully restored, which typically takes eight to 12 weeks. Activities are gradually resumed as tolerated by pain or soreness. Usually, it takes several weeks for complete healing [8]. Fractures can be classified on the following basis (Table 1).

**Table 1:** Ideburg classification [9]

Type	Description	Subtype
Type 1	Fractures of the rims	1A - Fracture of anterior rim
		1B - Fracture of posterior rim
Type 2	Fracture line that exits the scapula laterally through the glenoid fossa	
Type 3	Fracture line that exits the scapula superiorly through the glenoid fossa	
Type 4	Fracture line that exits the scapula medially through the glenoid fossa	
Type 5	Combinations	5A - Combination of types 2 and 4
		5B - Combination of types 3 and 4
		5C - Combination of types 2, 3, and 4
Type 6	Severe comminution	

Both operative and nonoperative scapular fracture treatment result in high union rates, high return-to-work rates, and minimal complications. Despite operative fixation resulting in no complications and restoration of anatomical function, Jones and Sietsema do not recommend surgery for scapula fractures with less than 20 mm displacement [10]. Further randomized prospective control studies with functional outcome data are required to further define indications for operative fixation.

## Conclusions

The primary objective was to medicate the avulsion fracture with conservative lines along with a ROM exercises to accentuate the healing along with strength training. Due to the rarity of these fractures, there is no distinct protocol for the management of such cases, and they are mostly based on the type of fracture. Inference of this particular case was conservative treatment being superior to operative treatment and conservative care of acromial fractures may be a preferred treatment until improved surgical techniques are discovered.
